# Isoelectric Point Separations of Peptides and Proteins

**DOI:** 10.3390/proteomes5010004

**Published:** 2017-01-25

**Authors:** Melissa R. Pergande, Stephanie M. Cologna

**Affiliations:** Department of Chemistry, University of Illinois at Chicago, Chicago, IL 60607, USA; mperga2@uic.edu

**Keywords:** isoelectric focusing, proteomics, electrophoresis, two-dimensional gel electrophoresis, isoelectric trapping, capillary isoelectric focusing

## Abstract

The separation of ampholytic components according to isoelectric point has played an important role in isolating, reducing complexity and improving peptide and protein detection. This brief review outlines the basics of isoelectric focusing, including a summary of the historical achievements and considerations in experimental design. Derivative methodologies of isoelectric focusing are also discussed including common detection methods used. Applications in a variety of fields using isoelectric point based separations are provided as well as an outlook on the field for future studies.

## 1. Introduction 

The separation of biomolecules, particularly proteins, in the presence of an electric field (e.g., electrophoresis) has given rise to an array of methodologies to reduce the complexity of samples to probe the physiochemical properties of such biomolecules. Proteins and peptides represent possibly the most highly studied class of molecules that are interrogated by electrophoretic methods. These methods include: agarose and polyacrylamide gel electrophoresis, two-dimensional gel electrophoresis (2DE), capillary electrophoresis, isotachophoresis and others.

**Figure 1:**
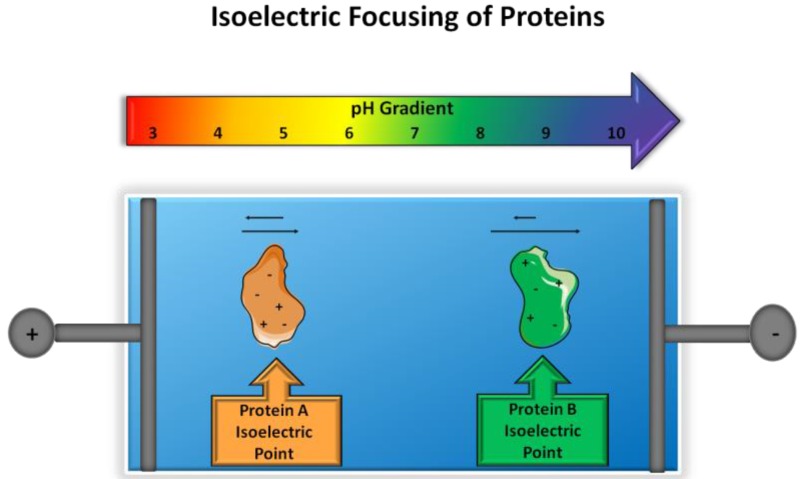
Principle of isoelectric focusing. Two proteins with varying isoelectric points will migrate in the presence of a pH gradient and electric field until the net charge of a protein is zero, in which migration will cease.

One such electrophoretic technique is isoelectric focusing (IEF) which provides separation of ampholytic components, molecules that act as weak acids and bases, according to their isoelectric points. In IEF, ampholytes travel according to their charge under the influence of an electric field, in the presence of a pH gradient, until the net charge of the molecule is zero (e.g., isoelectric point, pI). When considering peptides and proteins, the separation is deemed according to the composition of amino acids and exposed charged residues, which behave as weak acids and bases (Figure 1). The migration of the ampholytic species will follow basic principles of electrophoresis; however, the mobility will change in the presence of the pH gradient by slowing down migration at values close to the pI value. Even the simplest ampholytes (e.g., amino acids) can create a pH gradient and act as an isoelectric buffer.

The history of IEF begins with early work carried out by A.J.P. Martin [1] who made several contributions in the field of electrophoresis. Martin also contributed significantly to the field of chromatography and was awarded a Novel Prize for his efforts. The work of P.G. Righetti has been paramount in the ability to separate biomolecules electrophoretically, particularly according to isoelectric point. To fully understand these contributions, one must review the details of the experiment, particularly establishing the pH gradient. Furthermore, classical work regarding ampholytes was carried out by Svensson in the early 1960s [2,3,4].

Carrier ampholytes are the most commonly used chemical components used to generate pH gradients. The chemistry of carrier ampholytes was originally generated via pentaethylenehexamine and addition of acrylic acid. A second generation approach in carrier ampholyte synthesis was performed by Vesterberg [5], in which a heterogeneous mixture of amines was reacted with acrylic acid and a complex product resulted in the generation of thousands of molecules with varying pI values, yet very small changes in pI values across a pH range. Therefore, an ideal carrier ampholyte mixture is generated—a large number of components with close pI values resulting in a linear pH gradient. With regard to gels, carrier ampholytes can also be embedded into acrylamide gels and separation carried out in slab/flatbed format. Details regarding the specifics of carrier ampholyte synthesis and history have been previously reviewed [6,7].

A major achievement, which was an extension of the synthesis of carrier ampholytes, was the generation of immobilized pH gradients in 1982 [8]. Bjellqvist et al. utilized acrylamide as a backbone incorporating amino and carboxyl groups via radical mediated reactions allowing for branching and crosslinking with carrier ampholytes of different p*K*a values. The resulting product is a pH gradient that is immobile in an electric field and acts as a buffer. The values of pH range from 1 to 13 and can be synthesized in linear and nonlinear forms. The length of the IEF setup that is used plays a role in the desired resolution needed. This major advancement opened doors for various applications of isoelectric focusing for the separation of biological molecules, especially peptides and proteins. The resolving power of IEF (ΔpI) is determined by a series of factors in the experiment including the diffusion coefficient, conductivity and the electric current density. Properties of the gradient include the slope and the charge curve near the focusing point. These properties and relationships have been reviewed in detail [6,9].

IEF can be performed in a variety of formats, including preparative, analytical and microscale. On the larger end, IEF has proven to be beneficial as a preparative method due to its ability to separate large amounts of samples providing high resolution with large recovery yields. Notably, this separation method is advantageous in its ability to concentrate large quantities of samples while simultaneously removing common interfering agents or unwanted analytes. Additionally, IEF can be carried out in capillaries, microfluidic channels and multi-compartment electrolyzers (MCE) as described below. In general, IEF is an extremely powerful technique that, when used in any format, allows for the fractionation of samples resulting in reduced sample complexity and more in-depth analysis.

## 2. 2DE and IPG Strips

Building upon synthetic methods for carrier ampholytes, it was realized that coupling protein and peptide separation according to pI with other electrophoretic methods would be powerful. O’Farrell described in 1975 the combination of tubular IEF with sodium dodecyl sulfate-polyacrylamide gel electrophoresis separation in which radiolabeling was used for detection [10]. Additional related methods were also reported [11,12]. The report of immobilizing the pH gradient in 1982 [8] would provide further robustness of the method and provide a means for orthogonal and high resolution separation of proteins prior to downstream detection or characterization methods. Bjellqvist and colleagues report the linking of Immobilines™ to an acrylamide matrix to carry out IEF prior to a second dimension of separation [13,14]. Although this method proved to be beneficial in regards to it resolving power, the fragile tube gels lacked reproducible pH gradients. Perhaps one of the most significant advancements was the use of immobilized pH gradients (IPG) on plastic strips first introduced by Gorg and colleagues [15,16]. These strips are highly advantageous as pointed out by Westermeier, in that, after gel formation, all polymerization catalysts and unreacted compounds are washed away, providing the low electrical conductivity needed for IEF [17]. Furthermore, the more recent development of narrower gradient strips offers comparable resolution to longer strips of the same gradient. The use of immobilized pH gradients in strip format is now the standard procedure for carrying out 2DE [18,19]. Applications of 2DE span a broad range of disciplines and have been used for exploring the bacterial proteome [20], mapping protein phosphorylation sites [21,22] and expanding from two-dimensions. A report of a third dimension of electrophoresis has been used to eliminate co-migration challenges [23].

### 2.1. Capillary IEF (cIEF)

The development of carrier ampholytes provided the ability to perform solution-based IEF in a number of formats, with capillary separation being widely accepted. Building on the advantages of capillary electrophoresis, cIEF provides another dimension of separation in a small-volume format [24]. In cIEF, the gradient is generated across the capillary with the use of carrier ampholytes where the anode is immersed in an acidic solution and the cathode in a basic solution. Capillaries are coated at times; however, not all perform this step prior to electrophoretic migration. cIEF offers short separation times, high resolution separation and high peak capacity for small sample volumes. Separation of peptides by cIEF can be coupled with additional separation platforms or mass spectrometry [25]. Challenges arise after the separation, with regard to detection, as discussed later [26]. A series of secondary experimental procedures have been performed following cIEF. These include monolithic immobilized pH gradient columns in which carrier ampholytes are bound to the monolith within the column [27]. cIEF performed as a first dimension has been coupled with capillary electrophoresis experiments in which carrier ampholytes are removed and subsequently coupled with additional chromatographic methodologies [28]. 

### 2.2. Large-Format Devices

The use of carrier ampholytes to perform IEF separations has been carried out using several other types of devices beyond gel and capillary formats. The IsoPrime (Hoefer, GE, Little Chalfont, UK) was developed by Righetti and coworkers [29,30] to provide large-scale separations according to pI. The Rotofor [31] (Bio-Rad, Hercules, CA, USA) contains cylindrical focusing chambers with divisions into 20 compartments. Large amounts of proteins can be loaded (e.g., 1 mg) and the separation volume is upward of 55 mL. A smaller scale version of the Rotofor termed the “mini” is also available commercially. The mini Rotofor contains 18 mL of separation volume as compared to the larger device. It is important to note that, while the technique is not carrier ampholyte nor electrophoretic-based, chromatofocusing is another analytical method in which proteins or other ampholytes can be separated according to isoelectric point [32,33]. Typically, chromatofocusing utilizes ion exchange resins and changes in buffer pH to separate species according to their isoelectric point.

### 2.3. Small Scale Multi-Compartment Electrolyzers (MCE)

A number of small-scale IEF devices have been developed. The Zoom IEF device described by Speicher et al. [34,35] is a small scale MCE with acrylamide buffering membranes that can be used to perform solution-based IEF. Notably, Zoom IEF can also be classified as an IET device (described below) as it uses membranes with defined pH values and the addition of carrier ampholytes is optional. The Zoom IEF provided an appropriate means to fractionate ampholytes prior to downstream analysis. However, given that the device was made from plastic and had a fixed number of wells, the anode to cathode distance and maximal power load were drawbacks. Separations typically took hours albeit quicker than IPG methods. Other in-house developed devices have been described including a modification for two-dimensional separation [36], and Tran et al. reported a microscale, eight chamber device that was used for solution IEF separations on the time scale of one hour [37,38].

The OFFGEL Fractionator (Agilent Technologies, Santa Clara, CA, USA) is a hybrid-type MCE that also incorporates an IPG strip [39,40]. Fractionation is obtained via a two-phase system: the upper liquid phase is divided into wells with a lower phase consisting of an IPG gel strip and can be used to separate peptides or proteins. Ampholytes diffuse into the gel where the separation takes place on the strip. Then, fractions in solution are collected following the IEF process. Advantages of this technique include recovery of sample in the liquid phase, high resolution separation and little interference [41]. Those using this device have noted the long separation time needed, high amount of protein needed due to sample loss when fractionating proteins, poor resolution under non-denaturing conditions, and cathodic drift observed for proteins with alkaline pI values [41]. The majority of reports have utilized the OFFGEL device for peptide-based fractionation where it has been used for fractionation followed by liquid chromatography-mass spectrometry (LC-MS) analysis [42]. 

The smallest scale IEF separations are performed in microfluidic devices. Microfluidic devices provide advantages over more common analytical platforms owing to the small sample volumes and multiplexing capabilities [43,44]. Both carrier ampholyte and natural pH-gradient generation have been reported [45,46]. Coupling microfluidic separations, however, can prove challenging unless these methodologies are included in the microfluidic device. The small sample volumes can also result in difficulties in detection depending on the instrumentation being used. Righetti et al. proposed that the future for microchip electrophoresis lies within liquid polymer “sieving” capabilities [47]. Taken together, microfluidic IEF has been carried out for proteins [48,49] and peptides [50].

### 2.4. Isoelectric Trapping—IEF without Carrier Ampholytes

Unlike the generation of a continuous pH gradient as obtained commonly with the use of carrier ampholytes, separation according to pI without carrier ampholytes can be performed using buffering membranes only, termed isoelectric trapping (IET). In IET experiments, a series of buffering membranes are used to generate a step-wise pH gradient. The buffering membranes have been generated using an acrylamide backbone and co-polymerizing with carrier ampholytes (e.g., Immobilines). Acrylamide buffering members are extremely useful for such experiments and are used in small scale devices described above. However, it should be noted that acrylamide stability can be problematic at the extremes of pH (acidic and basic) and result in hydrolysis. As an alternative, poly(vinyl alcohol)-based (PVA) membranes were developed. PVA membranes are hydrolytically stable and have been used in a variety of IET experiments. The PVA membranes have tunable pH values based upon general acid-base chemistry. Other buffering membrane chemistries have been reported including agarose and poly(acryloylaminoethoxyethanol) [51]. 

**Figure 2:**
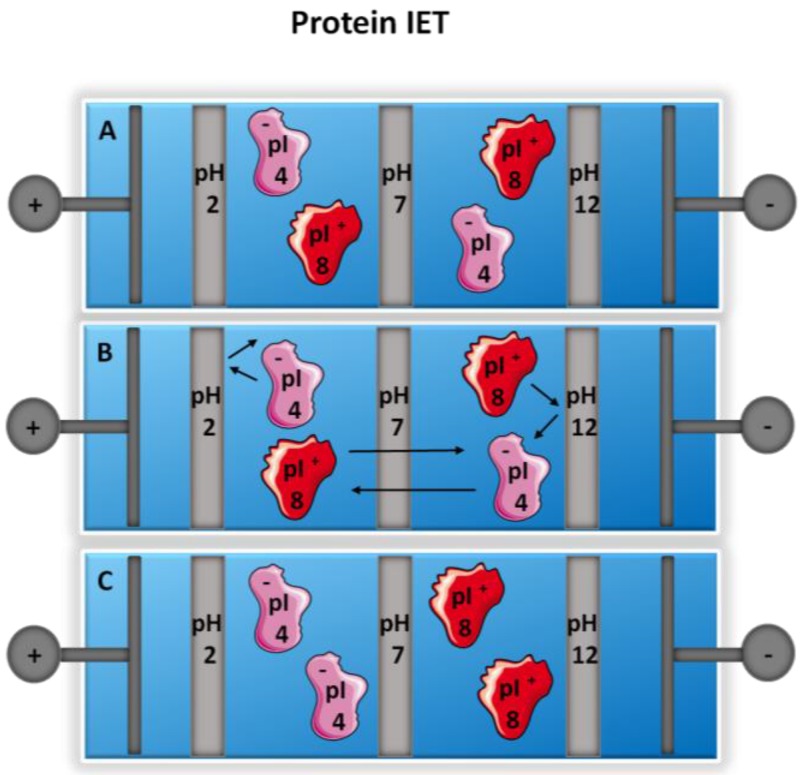
Isoelectric trapping (IET) of proteins. (**A**) Two proteins with differing isoelectric points are introduced into multi-compartment electrolyzer (MCE) assembled with pH buffering membranes; (**B**) the proteins migrate under the application of a potential and when the proteins reach a well in which two buffering membranes bracket the isoelectric point, the proteins are then trapped; and (**C**) migration continues between the two membranes, with continuous protonation and deprotonation of the protein. Adapted from references [54,55].

In IET, ampholytic components migrate according to their in-solution charge, which is in accordance with the amino acid sequence. Upon migration through the membranes into a well in which the pI falls between the pH values of the two adjacent buffering membranes, the ampholyte will then continuously migrate between the two buffers exchanging charge. Therefore, it is trapped (Figure 2). Given that the ampholyte is continuously charged, this reduces problems of solubility that are often observed when proteins are separated according to their pI where solubility remains low. To further address this, work has been done using pH-biasers added to the multi-compartment electrolyzer (MCE) to maintain pH and charge. Isoelectric buffers included have pI values that fall between the pH values of the two adjacent membranes. Common isoelectric buffers used in IET are single amino acids as well as synthetic buffers [52,53]. IET can be carried out in any MCE that contains buffering membranes. Lim et al. developed a small scale device termed “Membrane-Separated Wells for Isoelectric Focusing and Trapping” (MSWIFT) [54]. The MSWIFT device has a customizable number of wells that can be used for separation. Features of the MSWIFT include excellent heat dissipation, thereby having a high potential drop across the device and rapid separation. Various reports have highlighted the advantages of the MSWIFT device including desalting procedures, separation of small and large ampholytic components [54], bottom-up proteomic studies [55,56], and unique applications for purification [57]. Other IET devices have been constructed including the ConFrac [58,59] and TRECS [60,61] systems. It should be noted that any MCE that utilizes buffering membranes can be operated in IET mode. 

## 3. Detection of Peptides and Proteins

Following the separation of ampholytic components, one must consider how the resulting separation will be analyzed. This detection preference will also depend on the type of information that is needed as well as the format of separation. In gel-based separations such as IEF or 2DE, typically imaging is performed following the separation to visualize bands. From the image, one can then deduce and approximate the pI value and molecular weight of a band of interest. With regard to protein identification, the band is typically excised and mass spectrometry detection is performed. Capillary-based separations also suffer from challenges in detection, including consideration that ampholytes can be focused past the detector point. Pressure can be used post separation to mobilize the analytes across a detector. This can be ultraviolet (UV) detection where carrier ampholytes do not interfere or fluorescence detection can be used if fluorophores are present [62]. Detection can be obtained either at a single point or as full column imaging [63]. To address an issue of post-detector focusing, a blocker can be integrated into the capillary preventing the migration past the detector position. Salt can be used to mobilize focused ampholytes. Electrochemical detection has also been used [64]. Finally, in the majority of large-scale proteome studies currently being carried out, the mass spectrometer is used as the final detector. Therefore, consideration of compatibility must be included. 

## 4. Applications of Isoelectric Focusing and Related Methodologies

### 4.1. Protein Identification and Post-Translational Modifications

Protein identification from complex samples (e.g., cell and tissue lysates, plasma etc.) has been enabled by IEF. Given the orthogonal separations, in any format that IEF is used, it is possible to highly resolve complex samples allowing for deeper analysis of the proteome. Specifically, the power of 2DE can be realized when proteoforms are present, indicating modifications of the protein that can change the pI or molecular weight, information easily obtained with 2DE. There are reports of many examples demonstrating the power of 2DE in protein identification, characterization and determination of post-translational modifications, as recently reviewed [19]. Additional methodologies that incorporate IEF include separation of proteolytic digests using IPG strips [65]. The use of narrow-range IPG-IEF strips has been combined with nanoLC-MS in order to characterize the change in peptide pI and retention time due to various post-translational modifications [66]. Additionally, the use of high-resolution isoelectric focusing (HiRIEF) has recently been used for proteogenomic analysis, allowing for deeper proteome coverage and the discovery of new protein-coding loci [67]. Other applications include: analysis of sequential phosphorylation by protein kinases using fluorescent peptide substrates and microfluidic isoelectric focusing [68], monitoring protein folding dynamics [69] as well as two-dimensional approaches including the use of cIEF [70,71,72,73].

### 4.2. Separations for Mass Spectrometry Analysis

**Figure 3:**
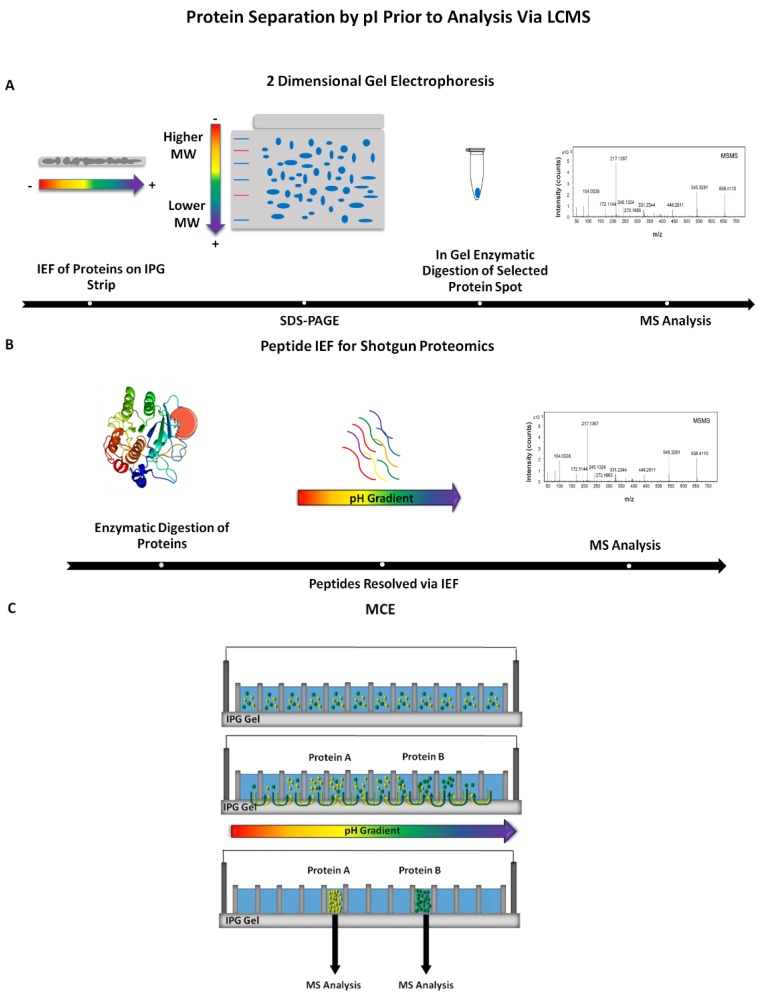
Examples of isoelectric focusing (IEF) methods incorporated prior to mass spectrometry (MS) detection. (**A**) Proteins are resolved by two-dimensional gel electrophoresis (2DE) and visualized with a stain. Spots are excised, subjected to proteolytic digestion and the resulting solution is analyzed via mass spectrometry; (**B**) Intact proteins, simple or complex mixtures, are proteolytically cleaved followed by the resulting peptides being fractionated according to pI. Separated peptides are then detected by mass spectrometry; (**C**) Separation of peptides or proteins in an MCE (i.e., OFFGEL) followed by analysis via mass spectrometry [39,40].

Perhaps the greatest power of 2DE and related IEF techniques is the complementarity with mass spectrometry. When used together, this approach is able to reduce proteome complexity with enough resolution that single proteoforms can be analyzed with far more depth compared to a standard shotgun approach. Figure 3 depicts several possible experimental IEF methodologies coupled with mass spectrometry. While the image obtained from the gel-based experiment provides information about the protein composition, complete determination of the protein in the absence of a standard is challenging. It is now common practice to perform 2DE in order to separate proteins and visualize spots while also obtaining quantitative information, followed by excision of the spot and downstream mass spectrometry analysis. Mass spectrometry plays a crucial role in analyte identification and characterization, particularly for peptide and protein analysis with the advancement in soft ionization techniques (e.g., matrix-assisted laser desorption/ionization (MALDI) and electrospray ionization (ESI)). 

Although there have been significant advances in regards to proteomic analysis via mass spectrometry, undersampling of the proteome and consequently the inability to obtain deep proteome coverage remains a problem when analyzing complex samples. For this reason, the use of multidimensional separation methods including IEF have been employed in order to reduce sample complexity prior to analysis. With the development of multidimensional protein identification technology (MudPIT, also termed shotgun), in which two-dimensional chromatography was carried out, extensive proteome coverage was obtained [74]. Following this development, a variety of alternative approaches were reported, many including the use of pI-based separations to fractionate prior to mass spectrometry analysis [75]. There are numerous reports in the literature that compare different separation methods that encompass IEF prior to tandem mass spectrometry. One of the most common orthogonal approaches used to resolve highly complex proteomes is the use of IEF-IPG and reverse phase liquid chromatography [76]. Another study showed that a three-dimensional IEF based approach provided superior resolution compared to previously reported 2D-gel electrophoresis results for the discovery of biomarkers when exploring the microbial metaproteomes in plants [77]. Interestingly, the use of high-pH reverse phase fractionation prior to mass spectrometry analysis has shown to result in a more uniform distribution of unique peptides across all fractions collected compared to off-gel IEF [78]. It is important to note, however, that interfering agents, found in common isobaric labeling kits (e.g., iTRAQ, Sciex, Framingham, MA, USA and TMT, TheroFisher Scientific, Waltham, MA, USA) cannot be removed via this method. Lengqvist and colleagues demonstrated that not only is IPG-IEF compatible with labeling techniques such as iTRAQ, but this separation method is also able to provide additional experimental data such as peptide pI in addition to high resolution fractionation [79]. Many of the above-mentioned techniques have been used in this approach for high resolution fractionation, protein identification and quantification by mass spectrometry.

### 4.3. Novel Approaches

There are many other novel approaches found in the literature that incorporate the use of IEF. For example, a unique methodology that incorporates IPG strips and MALDI mass spectrometry is the generation of a “virtual” 2D gel proteome. This is obtained by separation of proteins via isoelectric focusing in strip format followed by imaging mass spectrometry by MALDI [69]. Other electrophoretic methods including CE have been coupled to mass spectrometry [70,71] for a variety of proteomic applications including biomarker discovery in clinical specimens [72]. Complementary approaches have been described particularly in the development of new devices for electrophoretic separations. Herzog et al. described the generation of an on-chip reactor to fluorescently label proteins prior to separation according to charge [80]. In an effort to perform isoelectric point based fractionation without carrier ampholytes or isoelectric buffers, Chen and colleagues described a capillary method in which sample pH and the pH of the anolyte and catholyte solutions are tailored to perform fractionation steps according to charge and, therefore, pI [81]. The generation of dynamic pH profiles in an ampholyte-free separation has also been reported [82]. Related, tunable IEF has been reported by use of moving reaction boundary theory coupled with sodium dodecyl sulfate-polyacrylamide gel electrophoresis and resulted in an increase in protein spots and resolution [83]. Additionally, the use of microfluidic devices such as cIEF combined with online trypsin digestion, followed by analysis via nano reverse-phase LC-MS analysis [84], has proven to be advantageous in that few samples are needed, and it allows for the high-throughput analysis of low abundance proteins while reducing direct sample handling.

## 5. Conclusions

**Figure 4:**
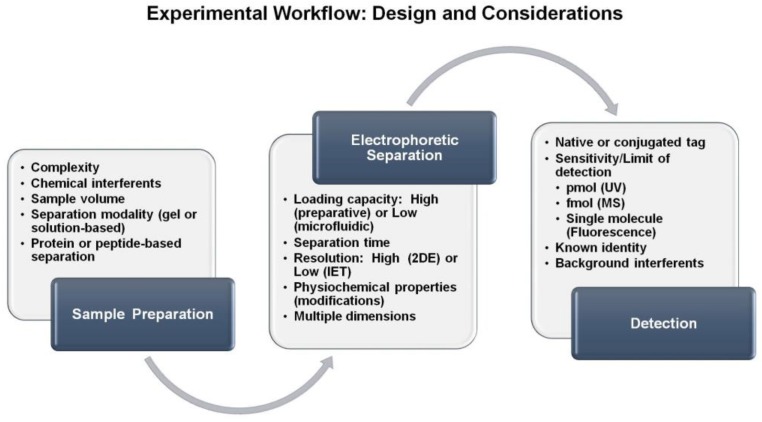
Experimental workflow summarizing important factors that should be considered when selecting an electrophoretic separation method for the analysis of protein and peptide samples.

Taken together, there are several steps to consider when using IEF for separating peptides or proteins. A general schematic of this workflow is provided in Figure 4 in which inherent properties are also included for the separation modality and detection modes. Once the sample has been identified, one must consider the necessary preparation steps prior to the electrophoretic separation. This may include buffer conditions that are suitable for electrophoresis, potential chemical interferents and whether the goal is to separate the peptide or protein component. Next, the separation technique should be determined. The selection of the separation technique will depend on a variety of factors including sample quantity, resolution desired and time needed to perform the separation. Furthermore, consideration of a second dimension of separation must be included. Finally, the detection of the analytes must be incorporated. If peptides or proteins are being detected, UV/Vis spectroscopy can be used, in which native absorbance relies on the peptide backbone and aromatic amino acids. Alternatively, more sensitive techniques such as fluorescence require the presence of a fluorophore that can be introduced in the sample preparation stage. If peptide or protein identification is needed, then coupling downstream with mass spectrometry would be the method of choice. The flexibility of the experimental design and compatibility with several analytical techniques makes isoelectric focusing an ideal method to include in biomolecule separation and characterization.

The development of isoelectric focusing to separate ampholytes according to isoelectric point has made significant impacts in the fields of separation science, mass spectrometry and proteomics. Regardless of format used, the benefits of this separation have been invaluable for peptide and protein studies. Given the advancements in the field of proteomics particularly incorporating mass spectrometry detection, the need for advanced separations will continue. The complexity of starting materials continues to be a problem in the analysis of peptides and proteins, and the interest in low abundance proteins and protein modifications are of great importance. Generation of a variety of devices to perform these electrophoretic separations is also expected to continue. While complexity is great, sample resources can also be limited. Therefore, small scale devices and microfluidics are likely to make significant contributions. In the past 40 plus years, impressive and significant achievements have been seen in electrophoresis and separation science. We will likely see continued growth in development in the future.

## Abbreviations

2DE	Two-dimensional gel electrophoresis
cIEF	Capillary isoelectric focusing
ESI	Electrospray ionization
HiRIEF	High-resolution isoelectric focusing
IEF	Isoelectric focusing
IET	Isoelectric trapping
IPG	Immobilized pH gradients Immobilized pH gradients
MALDI	Matrix-assisted laser desorption/ionization
MCE	Multi-compartment electrolyzers
MSWIFT	Membrane-separated wells for isoelectric focusing and trapping
pI	Isoelectric point
PVA	Poly(vinyl alcohol)
